# Giant, highly diverse protists in the abyssal Pacific: vulnerability to impacts from seabed mining and potential for recovery

**DOI:** 10.1080/19420889.2020.1843818

**Published:** 2020-11-25

**Authors:** Andrew J. Gooday, Jennifer M. Durden, Craig R. Smith

**Affiliations:** a National Oceanography Centre, Southampton, UK; b Life Sciences Department, Natural History Museum, London, UK; c Department of Oceanography, School of Ocean and Earth Science and Technology, University of Hawai‘i at Mañoa, Honolulu, HI, USA

**Keywords:** Xenophyophores, foraminifera, polymetallic nodules, habitat heterogeneity, ecosystem recovery

## Abstract

Xenophyophores, giant deep-sea agglutinated foraminifera, dominate the benthic megafauna in the eastern equatorial Pacific Clarion-Clipperton Zone. This abyssal (>4000 m depth) region hosts major deposits of polymetallic nodules targeted for future seabed mining, an activity that would destroy these highly diverse and delicate protists, particularly those living on the nodules themselves. Since the cell occupies only a small proportion of their test volume, xenophyophores may make a fairly modest contribution to benthic biomass and carbon cycling. Nevertheless, xenophyophore tests can passively enhance particle deposition, concentrate food, and provide habitat structure utilized by diverse organisms. Their destruction could therefore influence the recovery of benthic communities. Species requiring nodule substrates will likely not recover, since nodules take millions of years to form. However, xenophyophores can grow quickly and colonize extensive volcanic ash deposits within years, suggesting that sediment-dwelling species could be among the first large immobile organisms to reappear in mining-impacted areas.

## Introduction

Xenophyophores (Class Xenophyophoroidea), giant protists that build ‘tests’ composed of foreign particles obtained from the surrounding environment, are among the most enigmatic inhabitants of the deep seafloor. Their tests are morphologically very diverse and reach sizes of up to 24 cm, making them among the largest known single-celled organisms. Xenophyophores have a distinctive internal structure [1,2] and were formerly classified as a separate group of amoeboid organisms [2] until genetic analyses revealed them to be ‘monothalamous’ (single-chambered) foraminifera [3]. They are now firmly established as a monophyletic group forming one of the terminal branches of monothalamid Clade C [4].

Xenophyophores occur on both hard and soft substrates throughout the oceans at depths below about 500 m. Most are epifaunal [4–9] but infaunal tubular species are also known [10]. There is some evidence that these may form reticulated networks [10], although the suggestion that the net-like *Paleodictyon* structures found within in ancient and modern deep-sea sediments are xenophyophores [11] is not supported [12]. These protists are most common in habitats with an elevated supply of organic matter, for example, under upwelling areas, on seamounts, in submarine canyons, and other places where seafloor topography enhances current flow [2,6–8,13], although rare where currents are strong enough to mobilize the sediments and create active ripples [6,7]. They are also dominant and diverse members of the megafauna at the abyssal seafloor in the Clarion-Clipperton Zone (CCZ) [8,9], a huge swathe of the equatorial North Pacific between about 115° to 155°W that is characterized by very low currents. The CCZ hosts commercially important deposits of polymetallic nodules [14]. These potentially valuable resources are the focus of a nascent seabed mining industry, regulated by the International Seabed Authority (ISA), which requires [15] baseline biological surveys to be conducted in exploration contract areas of up to 75,000 km^2^. We hope that our brief review of key information about xenophyophores will encourage the inclusion of this important faunal component in future baseline studies and environmental impact assessments.

## Xenophyophore diversity in the CCZ and adjacent areas

Eighty-three species of xenophyophores were formally described from different oceans between 1883 and 2020; more than a third (35) occur in the equatorial Pacific to the east of 150° W, a region that includes most of the CCZ. The number of epifaunal xenophyophore species has been swelled by recent baseline surveys within the CCZ itself. This recent upsurge in xenophyophore research has focussed in the eastern CCZ, from where 20 new species and 3 new genera have been described [4,5,16–20]. A further 4 new species and 2 new genera were described recently in the western CCZ [4]. Of this total of 24 species, only two (*Stannophyllum radiolarium, S. zonarium)* are reported from outside the CCZ, although this likely reflects, to some extent, a lack of attention to xenophyophores in other parts of the Pacific rather than endemic distributions. A further 39 species have been recognized in collected material but are currently undescribed, bringing the CCZ total to 63 species (Table 1). More species undoubtedly await discovery.

**Table 1.** Described and undescribed xenophyophore species found within the Clarion-Clipperton Zone. N = species found attached to nodules; S = species found on sediment surface. Asterisks indicate species for which genetic data are available. Species names in bold refer to species recorded from outside the CCZ. Note that most species are rare and the absence of such records does not imply that species are endemic to the CCZ. All species are represented by published photographs. A few additional undescribed xenophyophores that were listed in publications [9,27,30] but not illustrated are omitted from the Table

	Reference	Substrate		Reference	Substrate
Described species			Undescribed species (continued)		
*Abyssalia foliformis	[4]	N	*Galatheammina sp. 3	9	S
*Abyssalia sphaerica	[4]	S	*Galatheammina* sp. 7	9	N
*Aschemonella aspera	[5]	N	*Galatheammina* sp. 8	9	N
*Aschemonella monilis	[5]	N, S	*Homogammina* sp.	9	N
*Aschemonella tubulosa*	[18]	S	*Occultammina* sp.	9	S
*Bizarria bryiformis	[19]	N	*Psammina* aff. *multiloculata*	9	S
*Cerelasma implicata*	[18]	N	*Psammina sp. 1	9	S
*Galatheammina interstincta	[19]	N	*Psammina sp. 2	9	N
*Moanammina semicircularis	[4]	N	*Psammina sp. 3	9, 20	N
*Psammina limbata*	[17]	N	*Psammina* sp. 4	9	S
*Psammina microgranulata	[20]	N	*Psammina* sp. 5	9	S
*Psammina multiloculata*	[17]	N	*Psammina* sp. 6	20	S
*Psammina rotunda	[20]	N	*Psammina* sp. B	4	S
*Psammina tenuis	[4]	S	*Psammina* sp. C	4	N
*Psammina tortilis	[20]	N	*P. aff. *limbata* form 1	20	S
*Semipsammina licheniformis*	[17]	N	*P*. aff. *limbata* form 2	20	N
*S. mattaeformis	[19]	N	*Reticulammina* sp.	4	S
*Shinkaiya contorta	[19]	S	*Rhizammina sp. 1	9	S
*Spiculammina delicata*	[16–18]	N	*?*Rhizammina* sp. 2	9	S
*Stannophyllum paucilinellatum*	[18]	N	*Semipsammina* sp. 5	9	N
***Stannophyllum radiolarium***	[17,31]	N	?*Shinkaiya* sp.^a^	9	S
*Stannophyllum setosum*	[32]	S	*Stannophyllum* sp.	17	N
*Stannophyllum zonarium	[4,9,31]	S	?*Syringammina* sp.	9	S
*Tendalia reteformis	[19]	S	*Xenophyophore sp. 1	9	S
			*Xenophyophore sp. 2	9	S
**Undescribed species**			Xenophyophore sp. 3	9	N
*Aschemonella* aff. *monile*	[4]	S	Xenophyophore sp. 4	9	S
*Aschemonella sp. 3	[9]	N	*Xenophyophore mudball	4	S
*Aschemonella* sp. 4	[9]	N	Indeterminate Xenophyophore	4	S
*Aschemonella* sp. 5	[9]	S	Irregularly anastomosing species	9	N
*Galatheammina* sp. 1a	[9]	N	Pale *Aschemonella-*like domes	9	N
*Galatheammina* sp. 1b	[9]	S	Pale patches	9	N
*Galatheammina sp. 2	[9]	?N			

^a^Misspelt (?*S**k**inkaiya*) in caption to Supplementary Fig. S12 in reference (9)

**Figure 1.**
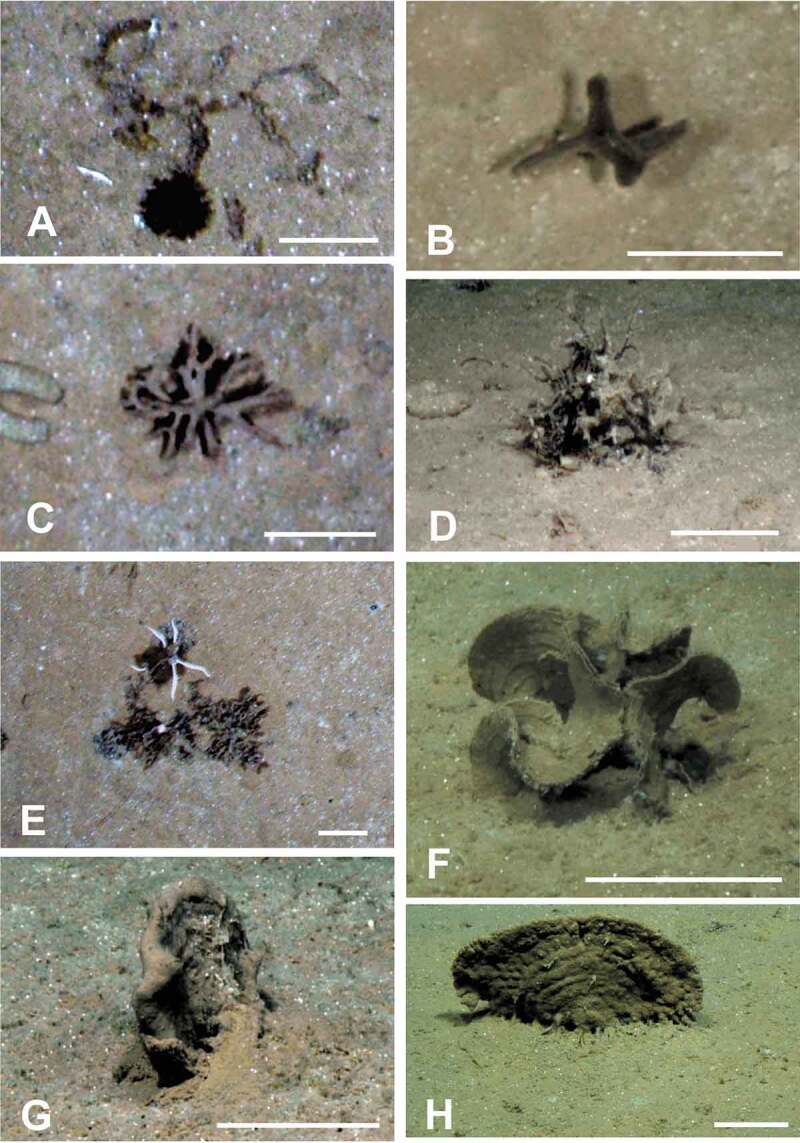
Seafloor images showing xenophyophores, or likely xenophyophores, taken from the ROV using the vertically mounted stills camera (A–C, E) and a forward-facing video camera (D, F–H). (A) Dark spiky sphere (possibly a xenophyophore) next to a branched, segmented tube (possibly a species of the xenophyophore genus *Aschemonella*); this is most likely a chance juxtaposition. APEI-1: 153.598° W, 11.251° N; 5204 m depth. (B) Dark, upright test with several branches; APEI-7: 141.896° W, 5.114° N; 4855 m depth. (C) Distinctive form comprising radiating branches; APEI-4: 149.939° W, 07.033° N; 5037 m depth. (D) Upstanding mass of branching tubes, possibly either *Aschemonella* or *Rhizammina*; APEI-1: 149.940° W, 07.036° N; 5040 m depth. (E) Irregularly-shaped patch with wrinkled surface, possibly a xenophyophore; shadows suggest that parts of the structure are raised above the sediment surface; APEI-4: 149.912° W, 06.990° N; 5003 m depth. Similar patches are common in the vertical images. Note the associated ophiuroid. (F) Test comprising a series of thin, curved plates with clearly-developed ‘growth lines’; APEI-4: 149.911° W, 07.009° N; 5018 m depth. Possibly a well-developed specimen of the recently-described species *Psammina tenuis* [4]. (G) Oblique view of relatively thick plate with vague ‘growth lines’; APEI-7: 141.816° W, 05.044° N; 4873 m depth. (H) Large plate-like xenophyophore with ‘growth lines’ and root-like structures anchoring it in the sediment; probably *Stannophyllum zonarium* [4]; APEI-1: 153.606° W, 11.252° N; 5206 m depth. Scale bars = 5 cm. Photo credits: Jennifer Durden and Craig Smith, DeepCCZ Project

**Figure 2.**
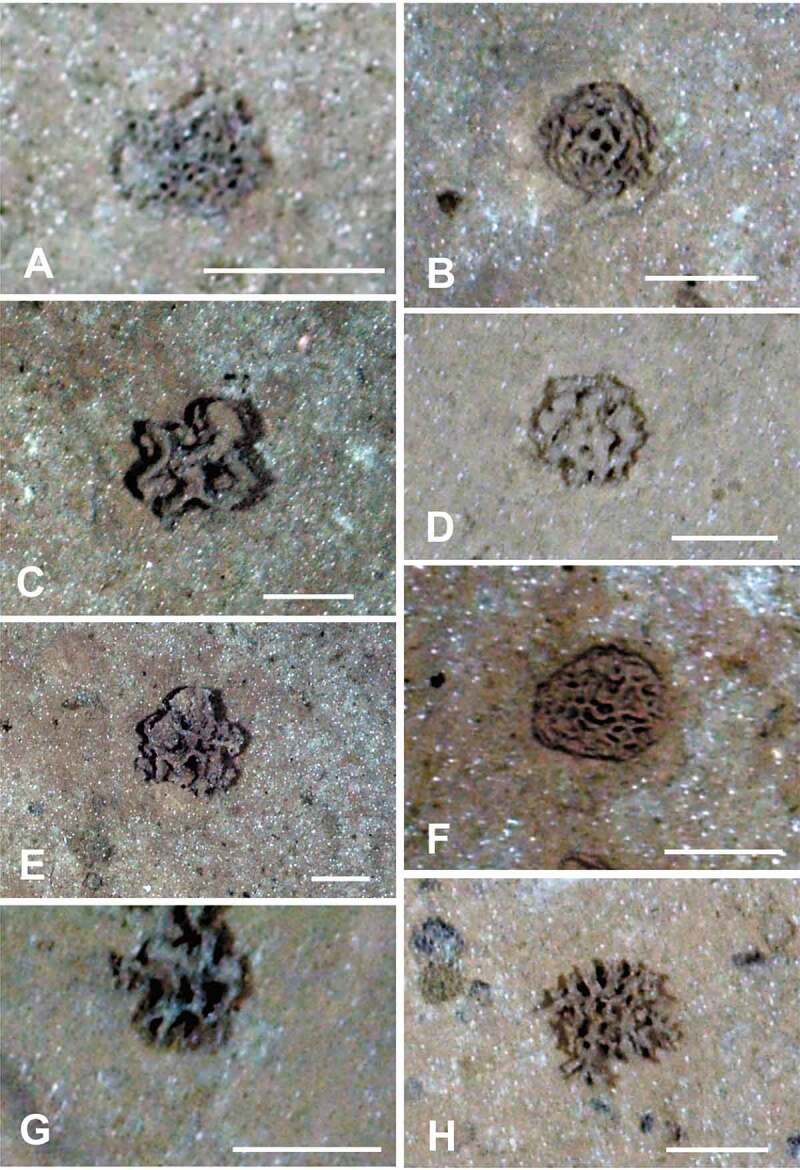
Seafloor images showing xenophyophores, taken from the ROV using the vertically mounted stills camera. (A) Finely reticulated test; APEI-7: 141.825° W, 05.056° N; 4870 m depth. (B) Reticulated dome, possibly a species of *Reticulammina*; APEI 7: 141.895° W, 05.114° N; 4855 m depth. (C) Test with thick, reticulated branches; APEI-7: 141.818° W, 05.048° N; 4873 m depth. (D) Dome with poorly-defined reticulations; rather similar to *Reticulammina* sp. of Gooday et al. (2020) [4]; APEI-1: 153.597° W, 11.251° N; 5204 m depth. (E) Test comprising irregular lamellate branches with a tendency to form reticulations; similar to C but with thinner branches; APEI 7: 141.822° W, 05.054° N; 4872 m depth. (F) Dome comprising thin, fairly densely-reticulated lamellae; APEI 7: 141.819° W, 05.049° N; 4873 m depth. (G) Irregular, coarsely-reticulated dome; APEI-4: 149.941° W, 06.973° N; 5007 m depth. (H) Test comprising reticulated branches or tubes; APEI 4: 149.938° W, 07.031° N; 5035 m depth. Scale bars = 5 cm. Photo credits: Jennifer Durden and Craig Smith, DeepCCZ Project

**Figure 3.**
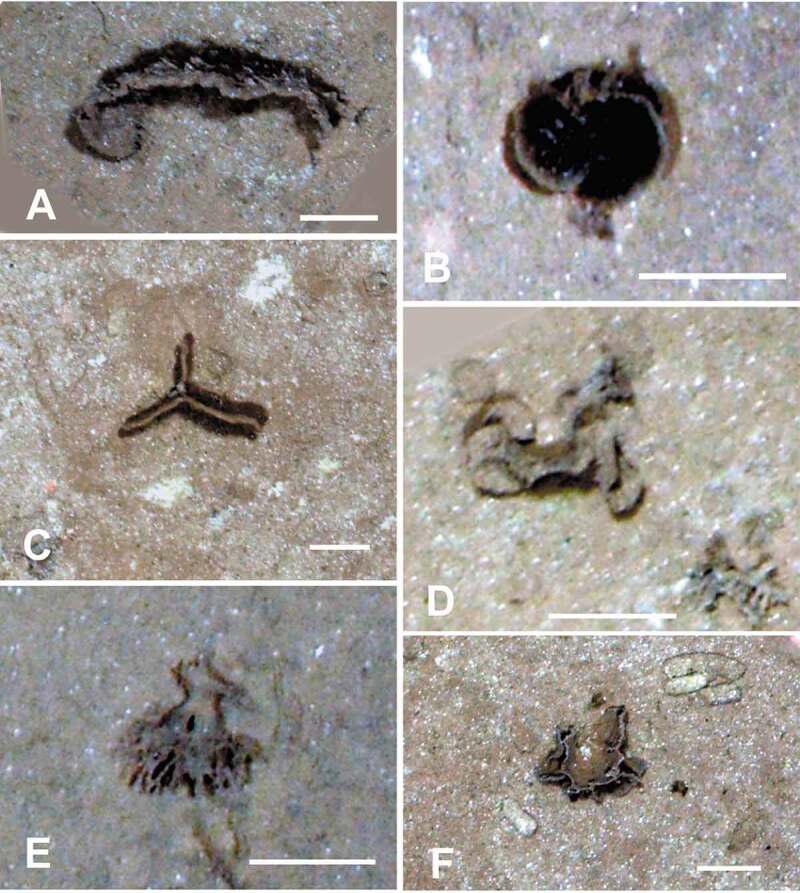
Seafloor images showing xenophyophores, taken from the ROV using the vertically mounted stills camera. (A) Curved plate embedded in the sediment; APEI 4: 149.912° W, 06.992° N; 5006 m depth. (B) Paired pale-rimmed plates; shadows indicate that these are raised above the sediment surface; APEI-1: 153.591° W, 11.251° N; 5200 m depth. (C) Vertically-orientated, triradiate plate, partly embedded in the sediment; APEI 7: 141.895° W, 05.114° N; 4855 m depth. (D) Test comprising rounded, plate-like elements; APEI 4: 149.938° W, 07.030° N; 5034 m depth. (E) Test with thick branched stem, dividing into narrower branches. Possibly disturbed from an originally upright position; APEI-1: 153.591° W, 11.251° N; 5199 m depth. (F) Thin ridge with side-branches arising from horizontal plate; APEI 7: 141.830° W, 05.059° N; 4868 m depth. Scale bars = 5 cm. Photo credits: Jennifer Durden and Craig Smith, DeepCCZ Project

Epifaunal xenophyophores are often the dominant megafaunal organisms visible in seafloor photographs across the CCZ [8,13,21,22,23]. Variation in test morphology, probably related to the optimization of food acquisition [6], is typical of many xenophyophores and can complicate the task of recognizing known morphospecies from images. Nevertheless, these images can provide additional information on xenophyophore diversity by revealing the presence of forms that have not been sampled physically. Two photographic surveys of megafauna in the eastern CCZ [8,21] distinguished 23 and 20 test morphotypes, respectively, all but one unidentified taxonomically. In the western CCZ, we have recognized at least 22 apparently distinct xenophyophore, or xenophyophore-like, morphotypes (Figures 1–3) in seafloor images, in addition to the 11 species described earlier [4]. Three of these (Figure 1f, 1h, 2d) are possibly the same as species that were collected [4], but the remaining 18 do not appear to be represented in the samples. These newly recognized morphotypes add to the known diversity of xenophyophores in the western CCZ. Together with earlier photographic surveys in the eastern half of the CCZ [8,13,21,23], they emphasize the extent of undescribed xenophyophore diversity within this nodule-rich region of the Pacific.

## Consequences of seabed mining impacts for xenophyophores

Seabed communities will suffer both direct and indirect impacts from seabed mining, including direct removal, habitat destruction, and burial/smothering from the redeposition of sediment suspended by mining [24]. Immobile organisms, including xenophyophores, will be particularly vulnerable [25,26]. Xenophyophores are also fragile, and more than half (~52%) of the 63 described and undescribed species that have been collected in the CCZ are sessile on nodules (Table 1). Most species have been collected too rarely to determine whether this is an obligatory lifestyle, although this appears likely in the case of stalked, fan-shaped xenophyophores that appear well adapted to suspension feeding [1], as well as flat, recumbent species encrusting nodule surfaces [17,27]. The nodules develop extremely slowly and it will be millions of years before this hard substrate is reestablished in mined areas [28]. The recolonization of abyssal plains by nodule-obligate xenophyophores will therefore occur only on geological time scales. Xenophyophores are common on rocky surfaces on seamounts located to the north and east of the CCZ [6], but there is currently no evidence that the numerous seamounts and abyssal hills within the CCZ itself provide refuges for nodule-obligate species [29].

Xenophyophores are by no means confined to hard substrates. They are common on soft sediments [33], and many of those seen in CCZ seabed imagery are not obviously associated with nodules (Figures 1–3). There is evidence that large xenophyophores can grow surprisingly quickly on soft sediments in some deep-sea habitats. Time-lapse photography recorded a tenfold increase in the volume of three xenophyophores over a 291-day period on the Madeira Abyssal Plain (NE Atlantic) [34]. These protists colonize biogenic mounds on equatorial Pacific seamounts [7] and in the CCZ [35], while in the South China Sea (2338–3322 m depth) a large xenophyophore species appeared on the surface of a volcanic ash layer within a decade of its deposition in 1991 [36], a process with some similarities to the redeposition of sediment from the plume of suspended material created by mining. Xenophyophores that do not require hard substrates might begin to recover from mining impacts on similar time scales through the spread of water-borne propagules resulting from sexual or asexual reproduction in other areas [37]. However, it is unclear to what extent observations can be extrapolated to the CCZ from other deep-sea settings where environmental conditions at the seafloor may be very different. Recolonization experiments would be a more direct approach to determining the nature of xenophyophore recolonization in the CCZ following disturbance, and what conditions might be required for this to happen.

The vulnerability of xenophyophores to extinction in the CCZ as a result of mining activities is difficult to assess. Species confined to small geographical areas will be more at risk than those that are widely distributed. Our recent study in the western CCZ yielded two species that are genetically identical to species found 3,800 km away in the eastern CCZ [4]. If ranges of this size are typical, the risk of complete extinction, rather than local destruction, would be minimal, particularly given the availability of refuges in the form of ‘Areas of Particular Environmental Interest’ (APEIs [38]), and possibly rocky surfaces. However, many xenophyophore species recently described from the CCZ are represented by only one or two specimens, making it impossible to say anything about their ranges.

## Ecological implications

Only a small proportion of the volume of a xenophyophore test is occupied by the branching cell body [1,39], and the cytoplasmic volume is further reduced by the accumulation of numerous, probably inert, intracellular barite crystals. It is also not normally possible to distinguish live from dead tests in seafloor photographs. Thus, the visual dominance by xenophyophores of the megafauna in seafloor images may not be matched by their contribution to benthic biomass [39]. They probably feed at a low trophic level, either by gathering material from the sediment surface, suspension feeding, trapping particles within complex test structures, or perhaps by taking up dissolved organic compounds [39], a purpose for which the extensively branching cell body [1] would be well suited. Their role in carbon cycling is still unquantified, although grazing traces on tests and studies of metazoan gut contents show that some animals feed on xenophyophores [39–41], indicating that they contribute to deep-sea food webs [42].

Of greater importance in potential mining areas may be the role that xenophyophore tests, whether alive or dead, play in concentrating organic matter and creating habitat heterogeneity [39]. On East Pacific seamounts, levels of ^234^Th activity in sediments within and beneath a xenophyophore test were three times higher than in a control core, an indication that the deposition of fine particles was enhanced around the test [43]. On the NW African margin, lipid analyses suggested that xenophyophore tests contain higher concentrations of labile compounds and bacteria than sediments [44]. In the same area, enhanced respiration in a sediment core containing a xenophyophore compared to a control core was attributed to the activity of associated microbes [45], or to the xenophyophore itself and/or associated microbes [39]. Genetic data showed that a xenophyophore from the Izu-Ogasawara Trench hosted a microflora that was different from that in nearby sediments [46]. Several studies have documented diverse meiofaunal and macrofaunal assemblages, and even fish eggs and embryos, occupying the exterior and interior of tests, as well as in the sediments beneath them [6,47,48], suggesting that xenophyophores may serve as refuges from predation and perhaps as nurseries, in addition to being sources of food [49]. Ophiuroids are among the most common large metazoans directly associated with xenophyophore tests (Figure 1(e)) [6]. They are sometimes seen coiled around the bases of xenophyophores [6], including in the CCZ [23], possibly because organic matter is concentrated there [39]. This association may explain the positive correlation observed in the eastern CCZ between the density of deposit-feeding ophiuroids and the abundance of xenophyophores [8]. Tracks of other megafauna (echinoids and scaphopods) are occasionally seen circling xenophyophore tests [6,50].

Given this range of interactions, the destruction of these large biogenic structures by mining would clearly have adverse consequences for the test-utilizing CCZ biota. If, as suggested above, xenophyophores that live on soft substrates are among the early recolonisers of mined or resedimented areas of seafloor, they could create new habitat heterogeneity relatively rapidly, compared to nodules growing at rates of millimeters per million years. This, and their ability to concentrate organic matter and possibly to enhance microbial activity, may assist the recovery of benthic communities in regions of the abyss that are likely to experience major disturbances from industrial activities in the fairly near future. It is notable that xenophyophores are among the organisms designated as indicators of Vulnerable Marine Ecosystems (VMEs) by the Food and Agriculture Organization of the United Nations [47], because they combine the potential to provide habitat structure with vulnerability to destruction by deep-sea demersal fishing. This activity may have impacts similar to those of deep-sea mining, albeit at shallower bathyal (<1500 m) depths.

## Seabed photography methods

Seabed imagery (Figures 1–3) was collected during the DeepCCZ Project cruise aboard the R/V *Kilo Moana* (expedition 1808; 14 May to 16 June 2018) on abyssal plains in three APEIs (numbers 1, 4 and 7) located in the western CCZ. These are protected areas, part of a system of nine such areas located along the length of the CCZ. All photography was conducted using a high definition forward-facing video camera and a vertically-mounted stills camera, both mounted on the *Lu’ukai* remotely-operated vehicle. Parallel lasers were used to provide a scale for seabed imagery.
